# Molecular Diversity Assessment Using Sequence Related Amplified Polymorphism (SRAP) Markers in *Vicia faba* L.

**DOI:** 10.3390/ijms131216457

**Published:** 2012-12-04

**Authors:** Salem S. Alghamdi, Sulieman A. Al-Faifi, Hussein M. Migdadi, Muhammad Altaf Khan, Ehab H. El-Harty, Megahed H. Ammar

**Affiliations:** 1 Legume Research Group, Plant production Department, Collage of Food and Agricultural Sciences, King Saud University, P.O. Box 2460, Riyadh 11451, Saudi Arabia; E-Mails: salfaifi@ksu.edu.sa (S.A.F.); h.migdadi@gmail.com (H.M.M.); altaf_sbs@yahoo.com (M.A.K.); ehabelharty@gmail.com (E.H.H.); ammarrice@gmail.com (M.H.A.); 2 College of science and humanity studies, Salman Ibn Abdulaziz University, P.O. Box 83, Alkharj 11492, Saudi Arabia

**Keywords:** *Vicia faba* L., genetic diversity, germplasm collection, SRAP markers

## Abstract

Sequence-related amplified polymorphism (SRAP) markers were used to assess the genetic diversity and relationship among 58 faba bean (*Vicia faba* L.) genotypes. Fourteen SRAP primer combinations amplified a total of 1036 differently sized well-resolved peaks (fragments), of which all were polymorphic with a 0.96 PIC value and discriminated all of the 58 faba bean genotypes. An average pairwise similarity of 21% was revealed among the genotypes ranging from 2% to 65%. At a similarity of 28%, UPGMA clustered the genotypes into three main groups comprising 78% of the genotypes. The local landraces and most of the Egyptian genotypes in addition to the Sudan genotypes were grouped in the first main cluster. The advanced breeding lines were scattered in the second and third main clusters with breeding lines from the ICARDA and genotypes introduced from Egypt. At a similarity of 47%, all the genotypes formed separated clusters with the exceptions of Hassawi 1 and Hassawi 2. Group analysis of the genotypes according to their geographic origin and type showed that the landraces were grouped according to their origin, while others were grouped according to their seed type. To our knowledge, this is the first application of SRAP markers for the assessment of genetic diversity in faba bean. Such information will be useful to determine optimal breeding strategies to allow continued progress in faba bean breeding.

## 1. Introduction

Faba bean (2*n* = 2*x* = 12 chromosomes) is characterized by its larger and more complex genome compared with other legumes. Its size of 13,000 Mb [1] is more than 10 times the size of soybean (*Glycine max*) (1200 Mb), four times the size of pea (*Pisum sativum*) (4000 Mb), and 29 times the size of alfalfa (*Medicago sativa*) (450 Mb) [2]. Due to the genome complexity, relatively little progress has been made in faba bean breeding for elite cultivars that can withstand adverse environmental conditions. Using molecular markers to assess genetic diversity is important not only for crop improvement efforts, but also for efficient management and conservation of plant genetic resources in genebanks. Several molecular markers have been used in the characterization of genetic diversity, gene tagging, and mapping of important genes in faba bean [3–7].

The genetic diversity of faba bean has been studied with a number of molecular marker systems, including restriction fragment length polymorphism (RFLP) [8,9] random amplified polymorphic DNA (RAPD) [9,10], amplified fragment length polymorphism (AFLP) [5,11,12], inter simple sequence repeats (ISSRs) [13,14], genomic microsatellites (SSRs) [15], and target region amplification polymorphism (TRAP) [16]. Molecular markers were used to study the taxonomic relationships between closely related species of *Vicia faba* L. by RFLP- and PCR-generated data [17] as well as analyze mitochondrial and chloroplast DNA [18–20]. In a previous study, long terminal repeat sequence retrotransposon based markers were compared for their usefulness in sequence specific amplified polymorphism (SSAP) marker development in two *Vicia* species (*V. narbonensis* and *V. faba* L.), which had distinguished between geographic origins of *V. faba* L. genotypes, but not between minor or major types [21].

Sequence-related amplified polymorphism (SRAP) is a simple and efficient molecular marker technique, reasonable throughput rate, disclosure of numerous co-dominant markers, ease of isolation of bands for sequencing, more reproducible than RAPDs and are easier to assay than AFLPs and, most importantly, targeting of open reading frames (ORFs) [22]. It has been used for genetic diversity and phylogenetic studies in different legume crops: lentil [23], alfalfa [24–26], pea [27]. The exploration of genetic diversity and the relationships among conserved faba bean germplasm collections is essential and of critical importance in establishing, managing, and ensuring the long-term success of faba bean breeding programs.

The aim of this study was to evaluate the potential of SRAP markers for assessing genetic relationships and diversity in faba bean germplasm collections. To our knowledge, this is the first application of SRAP markers for the assessment of genetic diversity among faba bean collections.

## 2. Results

A total of 55 different SRAP primer combinations using five forward and eleven reverse primers were evaluated for their ability to prime PCR amplification of eight randomly selected genotypes. Only fourteen primer combinations which showed consistently reproducible polymorphisms were selected and used to analyze all of the 58 faba genotypes. The features of the primers across all 58 genotypes tested are summarized in Table 1.

The 14 primer pairs generated a total of 1036 differently sized well-resolved peaks (fragments), of which all were polymorphic over all of the genotypes. In total, 10,700 data points (amplified fragments) were scored with an average of 764 peaks per primer pair across all genotypes, thereby confirming the high multiplex ratio expected for SRAPs. The capability of different primer pairs to generate SRAP fragments varied significantly, ranging from 10 in primer pair ME2/EM4 to 158 in primer pair ME1/EM2, with an average of 74 fragments per primer pair. The average number of fragments per primer pair ranged from 3 in primer ME2/EM4, to 31 in primer ME1/EM3, with an average of 13 fragments per primer per genotype.

**Figure 1:**
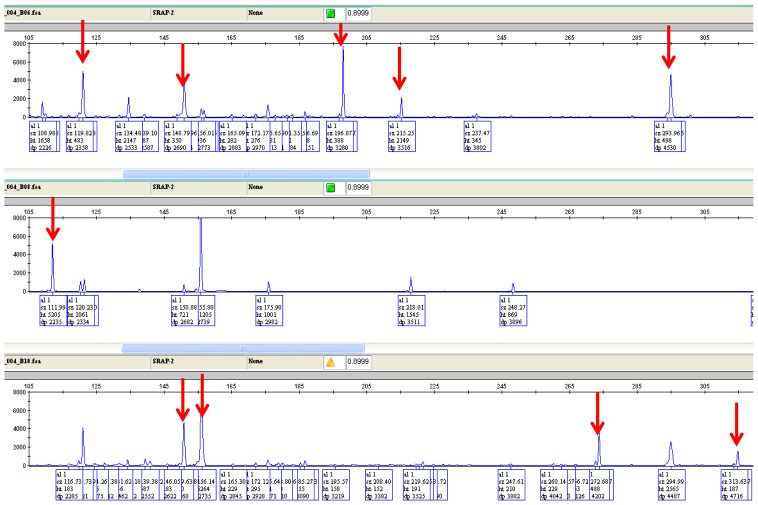
Electropherograms of three faba samples representing three genotypes using ME1/EM2 primers combination run on the Applied Biosystems 3130*xl* Genetic Analyzer displayed in the GeneMapper software v3.7 (Applied Biosystems: Foster City, CA, USA). The arrow denotes polymorphic peaks that are present or absent in just one sample.

All primer pairs generated 100% polymorphic fragments (Table 1). The polymorphic information content (PIC), measured as the percentage of polymorphic fragments for all primer pairs was high and varied in a relatively narrow range of 84% (for primer pair ME2/EM4) to 99% (for primer pairs ME1/EM2 and ME1/EM3), with an average of 96%. An example of a typical electropherogram representing the pattern of amplified DNA using all 58 faba genotypes with the polymorphic and common peaks is presented in Figure 1. Before the genotypes were used in analysis, the sizing quality, bin assignment, and allele calls were reviewed manually for accuracy. The genetic similarity estimates based on the Jaccard similarity coefficients for the SRAP data were used to assess the genetic relatedness among the 58 genotypes. The mean similarity indices presented by the genotypes ranged from 0.02 to 0.66 with an overall genotype similarity of 0.21.

**Figure 2:**
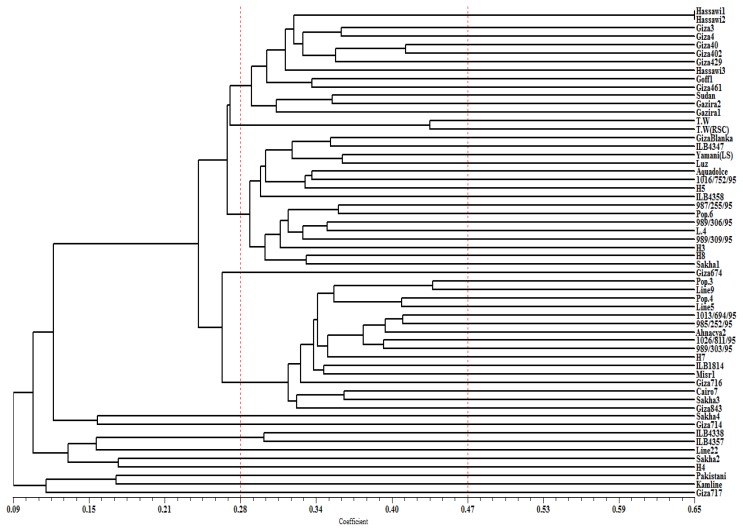
Dendrogram produced by Jaccard’s coefficient and the unweighted pair group method with arithmetic average (UPGMA) clustering method based on SRAP data in 58 faba bean genotypes.

The UPGMA cluster analysis of the genotypes based on the SRAP data was cut at a similarity of 0.28 (which represented 67% of the distance from the maximum similarity of 0.65 to the minimum of 0.09). Cutting the dendrogram at this similarity value resulted in three main clusters comprising 78% of the genotypes, two small clusters comprising 6% of the genotypes, and nine single clusters comprising 16% of the genotypes (Figure 2). The local genotypes (landraces) and most of the Egyptian genotypes, in addition to the Sudanese genotypes were grouped in the first main cluster with an average genetic similarity among these genotypes of 0.31. The closest genotypes were Hassawi 1 and Hassawi 2 with similarity index value of 0.65, while the most diverse genotype in this cluster was Gazira 1 with a 0.22 similarity value to the local genotype Goff 1 (Figure 2).

In the second main cluster, five elite breeding lines (produced from crosses utilizing the land races in the local faba breeding program at the legume research group) grouped with exotic genotypes with an average similarity index of 0.29. The closest genotypes in this cluster were Luz, Yamani (LS), and Pop.6, which had a 0.35 similarity index with Giza blanka, while the most diverse genotype in this cluster was sakha1 which had a 0.20 similarity value with Yamani (LS).

The third main cluster included an additional five advanced breeding lines produced from crosses utilizing the land races and Egyptian genotypes, with an average genetic similarity value 0.32. The advanced breeding lines (Pop.3 and line 9) were the most closely related with a similarity index value of 0.41, while Line 5 and Cairo 7 were the most distantly related in this cluster with the lowest similarity value of 0.26.

The two small clusters grouped Triple white (T.W) and Triple white (T.W) red seed coat in one cluster, and the two ICARDA’s genotypes (ILB4338 and ILB4357) in the second cluster. Nine genotypes formed individual clusters and were considered the most diverse genotypes. The dendrogram was further cut at 0.47, which represents 33% of the distance from the maximum similarity of 0.65 to the minimum of 0.09. Cutting the dendrogram at this similarity resulted in all of the genotypes forming separated clusters, with the exceptions of Hassawi 1 and Hassawi 2. At a similarity index of 0.66 Hassawi 1 and Hassawi 2 were separated to form two single clusters.

Group analysis of the genotypes according to their origin showed that cutting the dendrogram at a similarity value of 0.20 (which represented 25% of the distance from the maximum similarity of 0.26 to the minimum of 0.05) resulted in two main clusters, with the Pakistani genotype forming a third individual cluster.

**Figure 3:**
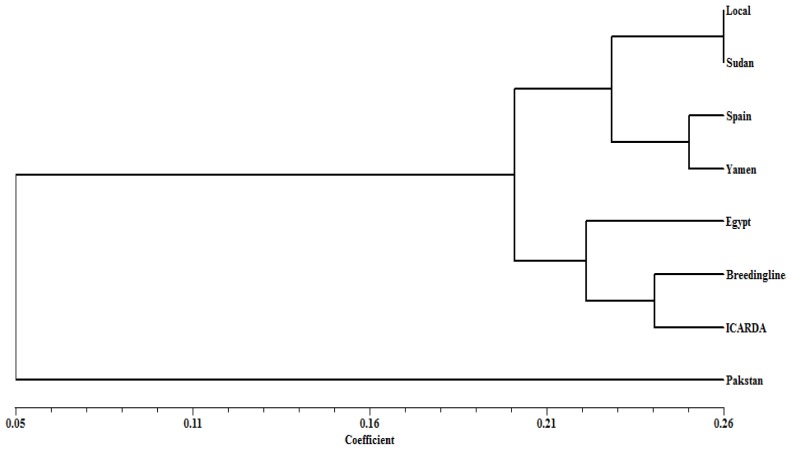
Dendrogram produced by Jaccard’s coefficient and the UPGMA clustering method based on SRAP data in eight faba bean genotypes resources.

The first cluster was further subdivided into two sub clusters. The first sub cluster included local landraces with Sudan genotypes, while the Yamani and Spain genotypes formed the second sub cluster. The national elite breeding lines clustered with breeding materials from ICARDA as well as the Egyptian genotypes, forming the second cluster (Figure 3).

## 3. Discussion

Assessment of the genetic variation within collections of faba bean genetic resources is crucial for the effective conservation and utilization of these resources in breeding programs, and could be dramatically enhanced by using molecular genotyping tools. Genetic variability in faba bean has been previously studied using different DNA molecular markers [3,8,10–16,28–31]. However this is the first application of SRAP markers as a tool for estimating genetic diversity in faba bean.

SRAP is a powerful technique for the assessment of genetic variability because it has shown a high degree of reproducibility and discriminatory power, as well as a high polymorphism rate in many genetic studies. In this study, the number of polymorphic fragments amplified by each primer averaged 74, ranging from 10 to 158 for all genotypes, with a range of 1 to 31 (average of 13) fragments per primer pair per genotype. All primer pairs generated 100% polymorphic fragments, so the number of fragments and the polymorphism percentages in this study were higher than those obtained in other genetic diversity studies utilizing SRAP markers on other plant species, or on faba bean using different molecular marker approaches [32]. Polymorphism rates and PIC values were used to measure the genetic diversity of faba genotypes in our collection. The average PIC value obtained in this study was 0.96, indicating that all of the SRAP markers showed high polymorphism and could contribute basic information to the genetics and breeding research of faba bean. It was recorded that 50 to 100 markers were enough to provide reliable pedigree information [33]. Nevertheless, it was reported that 150 polymorphic alleles were enough to estimate genetic similarities among maize inbred lines [34]. In this study, we analyzed 1,036 polymorphic fragments ranging in size from 100 to 500 bp, with an average of 74 fragments per combined primer pair. This was a relatively high number of SRAP markers compared to those obtained in other species [22,24,25,27,35–45], where the presence of 6–44 polymorphic bands per primer combination were reported.

The high polymorphism rate in this study coincides with those obtained in other plant species including 90% and 96.1% in alfalfa [24,25], 95% in buffalograss [36], 89% in jute [43], 87.59% in *Musa*[46], and 95.23% in *Paeonia*[47]. However, the polymorphic rate observed in this study was much higher than that generated using SRAP markers in other plant species, including 73.4% in cassava varieties [48], 56.0% in eggplant and related *Solanum* species [49], 47.2% and 72% in sesame [39,40], 81.97% in *Pogostemon*[50], 49.39% in *Brassica juncea*[51], 72.7% in *Cucurbita pepo*[52], 66.2% in *Cucurbita moschata*[35], 57% and 76.4% in safflower [41,42], 43% in *Coffea arabica*[45], 50% in Turkish okra [53], and 83% in sugarcane germplasm collections [37]. The variation of polymorphism rate reflects the extend of genetic divergence among and within the populations and/or genotypes studied and SRAP combinations used. These findings demonstrate the usefulness of SRAP markers in detecting of genetic variability in various plant species. The precision and accuracy in detecting genetic diversity at a molecular level makes it the marker system of the choice when studying closely related genotypes.

The high polymorphic rate (100%) and PIC value (0.96), together with the low genetic similarity (0.21) observed among genotypes in this study suggests a high level of heterogeneity, which is expected for faba bean since they are partially cross-pollinated and are heterogeneous mixtures of inbreds and hybrids, and is a result of outcrossing in faba bean [54–57]. In a previous study which utilized 364 AFLP fragments from five faba bean landraces, intra genetic variation ranged from 0.034 to 0.391 [58].

High levels of intra-population genetic diversity have been observed within the Mediterranean faba bean populations with the aid of ISSRs [13]. It was reported that the Mediterranean-type populations of faba bean are mixtures of *Vicia faba* L. minor, *Vicia faba* L. equine, and *Vicia faba* L. major [59]. This high Level of polymorphism in faba collections has been reported using different molecular markers, including ISSRs [13,14], AFLP markers [5,11,12,58], microsatellite markers [15], and TRAP markers [16]. However, [29] investigated the genetic diversity of faba bean from China and Europe using EST-SSR markers, and the results suggested that the genetic range of faba bean cultivars in China was narrow.

The UPGMA cluster analysis of the genotypes based on the SRAP data illustrated considerable association between the molecular diversity and geographic origin (source) of the genotypes. The local genotypes (landraces) and most of the Egyptian Giza genotypes, in addition to the Sudan genotypes were grouped in the first main cluster. A common feature of these genotypes is that they share a seed type, which is the equine type. Additionally, Hassawi 2 was used as one of the parents in the production of the Gazira 1 and Gazira 2 genotypes. The second cluster encompassed genotypes from Yemen and Spain, which have the *Vicia faba* L. major seed type. The KSU advanced breeding lines were scattered in the second and third main clusters with breeding lines from the ICARDA and genotypes introduced from Egypt, which suggests that these advanced lines were probably developed by utilizing ICARDA and Egyptian faba germplasm.

The T.W and T.W red seed coat were grouped in one small cluster, where T.W red seed coat is a mutant derived from T.W with a different seed coat color. ILB4338 and ILB4357, ICARDA’s breeding lines, were grouped in the second small cluster, suggesting that these genotypes could have the same genetic background. The genotypes singled out of these clusters are considered the most diverse genotypes, and they also have distinctive phenotypic traits. The Pakistani genotype is characterized by its dark seed coat color, minor type, and short plant height. The Kamline genotype has a white seed coat color with equine type, and the Sakha 2 genotype has the *Vicia faba* L. major type seed coat. Furthermore, group analysis of the genotypes according to their geographical origin showed that the Pakistani genotype, which formed an individual cluster, was the most diverse.

Our findings coincide with those of [5], who reported that the Asian faba lines clustered separately from lines from other geographic regions including those from Northern Europe, Southern Europe, and North Africa using AFLP markers. It was also confirmed that faba bean entries collected from within China formed a separate distinct group from faba bean collected from Europe, Africa, and other parts of Asia [11,12]. Our results were in partial agreement with those of [27], which used SRAP markers for characterization of pea accessions, and found no relationship between the origin of the accessions and the morphological and molecular clusters reflecting the exchange of germplasm among breeding programs in different countries.

Our results with SRAP markers suggest that the collection of faba bean cultivars utilized in this study is genetically diverse, in contrary to the results published by [29] who utilized EST-SSR markers. The clustering results of the studied genotypes were largely dependent on seed type rather than geographical origin. The clustering based on seed type is quite logic since *major*, *minor* and *equine* seed types represents the main cultivars groups [60] or even interfertile subspecies in some classification schemes of *Vicia faba* L. [61]. Thus, the clustering was clearly coherent with genetic constitution rather than origin *per se*.

## 4. Experimental Section

### 4.1. Plant Materials and DNA Extraction

Two week old faba bean leaves from 58 selected genotypes (Table 2) were collected, dropped in liquid N_2_, and stored at −80 °C until DNA isolation could be performed. DNA isolation was carried out using a modified SDS protocol [62].

The samples were ground in liquid N_2_ and 200 mg were mixed with 800 μL of extraction buffer (100 mM Tris-HCl pH 8, 50 mM EDTA pH 8, 1.4 M NaCl, 2% SDS *v*/*v*, PVP 2% *v*/*v*, and 0.1% mercaptoethanol), and incubated at 65 °C for 30 min. Then, 3 μL RNase1 (10 mg/mL) was added to each sample and incubated at 37 °C for 15 min. An equal volume (100 μL) of chloroform-isoamyl alcohol 24:1 was added; the samples were mixed well, and centrifuged at 13,680× *g* for 20 min. The supernatant was transferred to new 1.5 mL tubes and a 1/3 volume of 5 M potassium acetate was added. The samples were vigorously mixed, and centrifuged at 13,680× *g* for 20 min. The supernatant was removed and transferred to new 1.5 mL tubes, ½ volume of cold isopropanol was added, and the samples were mixed well and incubated at 4 °C for 1 h. The samples were then centrifuged at 13,680× *g* for 15 min at 4 °C. The supernatant was poured off and the tubes were inverted and allowed to air dry for 10 min. The pellets were re-suspended in 300 μL of TE (10 mM Tris, 1 mM EDTA, pH 8.0), incubated at 65 °C for 30 min, and the samples were centrifuged at 13,680× *g* for 5 min at 4 °C. The supernatants were transferred to new 1.5 mL microfuge tubes, and 1/10th volume of 3 M sodium acetate and 2/3rd volume of ice-cold isopropanol were added. The samples were mixed well, incubated at 4 °C for 1 h, and centrifuged at 13,680 × *g* for 10 min at 4 °C to pellet the DNA. The solution was discarded and the pelts were washed with 80% EtOH for 10 min, centrifuged at 13,680× *g* for 10 min at 4 °C, the solution discarded, and the tubes were inverted to dry for 30 min. The DNA samples were dissolved in 100 μL of TE and kept at 4 °C overnight. The quality and concentration of the extracted DNA was detected using a spectrophotometer. Dilutions with TE were carried out and concentration was fixed at 100 ng/μL.

### 4.2. SRAP-PCR

Fifty five SRAP primer combinations (5 forward and 11 reverse) were tested for their ability to prime PCR amplification of 8 selected faba genotypes. Fourteen SRAP primer combinations that showed consistently reproducible polymorphisms were selected and used to analyze all of the 58 faba genotypes (Tables 1 and 3). The forward primers were 5′ end labeled with FAM dye. The PCRs were performed in 20 μL reaction volumes containing 1× *GoTaq* Green Master Mix (Cat. No. M7123, Promega Corporation, Madison, WI, USA), 0.1 μM of each forward and reverse primer, 50 ng DNA template, and nuclease-free water to 20 μL. The thermal cycler profile for PCR amplification was set on a TC-5000 thermal cycler (Bibby Scientific, Staffordshire, UK) as follows: denaturation at 94 °C for 5 min, followed by five cycles of denaturing at 94 °C for 1 min, annealing at 35 °C for 1 min, and elongation at 72 °C for 1 min. In the remaining 30 cycles, the annealing temperature was increased to 50 °C for 1 min followed by a final elongation step at 72 °C for 7 min. For electrophoresis, 1 μL of the PCR amplified product was mixed with 0.5 μL of the GeneScan 500 LIZ size standard (Applied Biosystems P/N 4322682), and 8.5 μL of Hi-Di Formamide (Applied Biosystems P/N 4311320). The mixture was denatured and loaded on the 16-capillary system of the Applied Biosystems 3130*xl* Genetic Analyzer. A 36-cm capillary array (Applied Biosystems P/N 4315931) and 3130 POP-7 polymer (Applied Biosystems P/N 4352759) were used.

### 4.3. Data Scoring and Statistical Analysis

SRAP fragment analysis was performed with the *GeneMapper* Analysis Software v3.7 (Applied Biosystems: Foster City, CA, USA), and the data were assembled in binary format (allele presence (1) or (0) for absence). The threshold for fragment calling was set at 200 relative fluorescence units (rfu) [63], so that any peaks at 200 rfu or higher was assigned a 1 and those that were lower were assigned a 0. Fragment analysis was carried out for fragment sizes in the range of 100–500 bp. Fragments that were amplified in less than three genotypes were eliminated from the analysis. The polymorphism information content (PIC) for each primer was calculated to estimate its allelic variation according to the formula

$$\text{PIC}=1-\sum_{j=1}^{n}{P_{ij}}^{2}$$

Where *P*_ij_ is the frequency of the *i*^th^ allele for marker j and the summation extends over *n* alleles, calculated for each SRAP marker [64]. Data generated from SRAP analysis were analyzed using Jaccard similarity coefficient [65]. These similarity coefficients were used to construct dendrogram using the unweighted pair group method with arithmetic average (UPGMA) employing the SAHN (sequential, agglomerative, hierarchical, and nested clustering) from the *NTSYSpc* (version 2.10) program [66].

## 5. Conclusions

The rich polymorphism rate (100%) and low genetic similarity (0.21) indicated the high genetic diversity and broad genetic basis of our collection. The large number of polymorphic amplified fragments produced in this study (1036), with an average of 74 fragments per primer pair indicates that this system is a reliable and powerful tool to evaluate genetic polymorphisms and relationships among faba bean genotypes. Such information will be useful to determine optimal breeding strategies, and to allow continued progress in faba bean breeding. Diverse genetic backgrounds among parental lines provide a large supply for allelic variations that can be used to develop new favorable gene combinations in faba breeding programs.

## Figures and Tables

**Table 1:** The features of Sequence-related amplified polymorphism (SRAP) primers selected in faba bean genetic diversity.

Primer combination	Total fragments a	Average fragments b	Total no. of fragments c	PIC value
ME1/EM1	85	18	1,047	0.97
ME1/EM2	158	26	1,563	0.99
ME1/EM3	134	31	1,817	0.99
ME1/EM4	56	11	662	0.97
ME2/EM1	46	8	444	0.95
ME2/EM2	108	24	1,417	0.98
ME2/EM4	10	3	190	0.84
ME3/EM1	77	17	1,013	0.97
ME3/EM2	69	7	414	0.97
ME3/EM3	66	5	323	0.98
ME3/EM4	54	10	567	0.96
ME4/EM2	59	5	323	0.97
ME4/EM3	16	4	220	0.92
ME4/EM4	98	12	700	0.98
Total	1,036	-	10,700	-
Average	74	13	764	0.96

a Total number of differently sized SRAP fragments amplified across all 58 genotypes;

b Average number of SRAP fragments scored per genotype;

c Total number of SRAP fragments scored for all genotypes.

**Table 2:** Name, origin, and seed type of faba bean genotypes used in the study.

Entry No.	Entry name	Origin	Seed type
1	Hassawi1	KSA	Equine
2	Hassawi2	KSA	Equine
3	Hassawi3	KSA	Equine
4	Goff1	KSA	Equine
5	T.W.(red seed)	KSA	Equine
6	H4	KSA	Equine
7	H7	KSA	Equine
8	Line 9	KSA	Equine
9	Line 5	KSA	Equine
10	Line 22	KSA	Equine
11	Pop.6	KSA	Equine
12	H3	KSA	Equine
13	H5	KSA	Equine
14	H8	KSA	Equine
15	L. 4	KSA	Equine
16	Pop. 3	KSA	Equine
17	Pop. 4	KSA	Equine
18	Giza 3	KSA	Equine
19	Giza 4	Egypt	Equine
20	Giza 40	Egypt	Equine
21	Giza 402	Egypt	Equine
22	Giza 429	Egypt	Equine
23	Giza 461	Egypt	Equine
24	Gizablanka	Egypt	Major
25	1013/694/95	Egypt	Equine
26	1026/811/95	Egypt	Equine
27	985/252/95	Egypt	Equine
28	989/303/95	Egypt	Equine
29	Misr 1	Egypt	Equine
30	Sakha 2	Egypt	Major
31	Sakha 3	Egypt	Equine
32	Giza 716	Egypt	Major
33	Giza 717	Egypt	Equine
34	Giza 843	Egypt	Equine
35	1016/752/95	Egypt	Equine
36	987/255/95	Egypt	Major
37	989/306/95	Egypt	Major
38	989/309/95	Egypt	Major
39	Sakha 1	Egypt	Equine
40	Sakha 4	Egypt	Equine
41	Giza 674	Egypt	Equine
42	Giza 714	Egypt	Equine
43	Cairo 7	Egypt	Equine
44	ILB 4338	ICARDA	Equine
45	ILB 4357	ICARDA	Equine
46	ILB 1814	ICARDA	Major
47	Ahnacya 2	ICARDA	Equine
48	ILB 4347	ICARDA	Equine
49	ILB 4358	ICARDA	Major
50	Pakistani	Pakistan	Minor
51	Luz	Spain	Major
52	Aquadolce	Spain	Major
53	Kamline	Spain	Minor
54	Sudan	Sudan	Equine
55	Gazira 1	Sudan	Major
56	Gazira 2	Sudan	Minor
57	T.W.	Sudan	Equine
58	Yamani(Large seed)	Yemen	Major

KSA, Kingdom of Saudi Arabia; ICARDA, International Center for Agricultural Research in Dry Areas.

**Table 3:** Name and sequence of the SRAP primer used in faba genotypes screening.

Forward primers	5′→3′	Reverse primers	5′→3′
ME1	TGAGTCCAAACCGGAA	EM1	GACTGCGTACGAATTAAT
ME2	TGAGTCCAAACCGGAC	EM2	GACTGCGTACGAATTTGC
ME3	TGAGTCCAAACCGGAT	EM3	GACTGCGTACGAATTGAC
ME4	TGAGTCCAAACCGGAC	EM4	GACTGCGTACGAATTTGA
